# Differential intra-host infection kinetics in *Aedes aegypti* underlie superior transmissibility of African relative to Asian Zika virus

**DOI:** 10.1128/msphere.00545-23

**Published:** 2023-11-09

**Authors:** Rinyaporn Phengchat, Phonchanan Pakparnich, Chatpong Pethrak, Jutharat Pengon, Channarong Sartsanga, Nunya Chotiwan, Kwanchanok Uppakara, Kittitat Suksirisawat, Louis Lambrechts, Natapong Jupatanakul

**Affiliations:** 1 National Center for Genetic Engineering and Biotechnology (BIOTEC), Khlong Luang, Pathum Thani, Thailand; 2 Chakri Naruebodindra Medical Institute, Faculty of Medicine Ramathibodi Hospital, Mahidol University, Samut Prakan, Thailand; 3 Institut Pasteur, Université Paris Cité, CNRS UMR2000, Insect-Virus Interactions Unit, Paris, France; University of Saskatchewan, Saskatoon, Saskatchewan, Canada

**Keywords:** arbovirus, Zika, midgut infection barrier, transmission, infection kinetics

## Abstract

Despite numerous studies highlighting the higher transmissibility of the African Zika virus (ZIKV) lineage compared to the Asian lineage in mosquito vectors, little is known about how the viruses interact with different tissues during the early steps of mosquito infection. To address this gap, we aimed to characterize intra-host infection barriers by combining tissue-level monitoring of infection using plaque assays and a novel quantitative analysis of single-cell-level infection kinetics by *in situ* immunofluorescent staining. Our results revealed that, in *Aedes aegypti*, an African ZIKV strain exhibited a higher replication rate across various tissues than an Asian ZIKV strain. This difference was potentially due to a higher virus production in individual cells, faster spread within tissues, or a combination of both factors. Furthermore, we observed that higher blood meal titers resulted in a faster viral spread to neighboring cells suggesting that intra-host infection dynamics depend on inoculum size. We also identified a significant bottleneck during midgut infection establishment for both ZIKV lineages, with only a small percentage of the virus population successfully initiating infection. Finally, the *in situ* immunofluorescent staining technique enabled the examination of virus infection characteristics in different cell types and revealed heterogeneity in viral replication. Together, these findings demonstrate that differences in intra-host infection kinetics underlie differential transmissibility between African and Asian ZIKV lineages. This information could serve as a starting point to further investigate the underlying mechanisms and ultimately inform the development of alternative control strategies.

**IMPORTANCE:**

The recent Zika virus (ZIKV) epidemic in the Americas highlights its potential public health threat. While the Asian ZIKV lineage has been identified as the main cause of the epidemic, the African lineage, which has been primarily confined to Africa, has shown evidence of higher transmissibility in *Aedes* mosquitoes. To gain a deeper understanding of this differential transmissibility, our study employed a combination of tissue-level infection kinetics and single-cell-level infection kinetics using *in situ* immunofluorescent staining. We discovered that the African ZIKV lineage propagates more rapidly and spreads more efficiently within mosquito cells and tissues than its Asian counterpart. This information lays the groundwork for future exploration of the viral and host determinants driving these variations in propagation efficiency.

## INTRODUCTION

Zika virus (ZIKV) is a mosquito-borne flavivirus primarily transmitted from mosquitoes to humans through bites of infected *Aedes* mosquitoes. First identified in Uganda in 1947 from a sentinel monkey, sporadic ZIKV cases have been reported in Africa and Asia over the following decades (1, 2). ZIKV spread to several Pacific islands between 2013 and 2014 and reached South America in 2015, causing an outbreak that affected hundreds of thousands of people, underlining the potential of emerging and re-emerging arthropod-borne (arbo) flavivirus outbreaks (2). Phylogenetic analysis of ZIKV genome sequences revealed two major ZIKV lineages, the African and the Asian lineage, with studies indicating that the African lineage is more transmissible by mosquito vectors than the Asian lineage (3–5). However, the complex infection kinetics of ZIKV in mosquitoes and its interactions with individual tissues prior to transmission remain poorly understood.

Following ingestion of an infectious blood meal by mosquito vectors, the viruses must propagate in different body compartments and eventually be secreted in mosquito saliva when taking subsequent blood meals for transmission to occur. Several anatomical bottlenecks must be overcome for successful virus transmission in insects, including midgut infection, midgut escape, dissemination, salivary gland infection, and salivary gland escape barriers (6). Traditional techniques for studying virus infection kinetics, such as quantitative RT-PCR or infectious virus titration with cell-based assays, involve tissue grinding or lysis to extract and quantify viral RNA or infectious particles. While these methods can provide information on infection level, they have a significant limitation: the loss of spatial information on the infection kinetics. By contrast, *in situ* immunofluorescent staining preserves the spatial information of virus infection. Although this technique has the potential to provide a more comprehensive overview of infection kinetics including the sites of initial infection, cell-type tropism, rate of virus propagation, and spread within the tissues, its application has remained limited to investigate arbovirus-mosquito infection.

In this study, we infected *Aedes aegypti* (*Ae. aegypti*) mosquitoes with African (DAK AR41524) or Asian (SV0010/15) ZIKV and investigated intra-host infection kinetics at the tissue level by virus titration, and at the single-cell level using immunofluorescent staining. The results provide evidence that the African strain ZIKV has superior infectivity from the establishment of midgut infection, the spread of infection in the tissue to the replication at the cellular level. Our data demonstrate that the detailed analyses of *in situ* immunofluorescent staining offer a powerful tool to compare steps that determine differential infectivity of arboviruses in the insect vector.

## MATERIALS AND METHODS

### Animal maintenance

A laboratory strain of *Ae. aegypti* obtained from the Department of Medical Sciences, National Institute of Health Thailand and further maintained at BIOTEC’s insectary for an additional 36–38 generations was used for all the infection studies. Mosquitoes were maintained at 28 ± 1°C with 70% relative humidity and a 12-hour day/night, 30-minute dusk/dawn lighting cycle. The larvae were fed on powdered fish food (Tetra Bits). Adults were fed on a sterile 10% sucrose solution. To obtain the eggs for colony maintenance, mosquitoes were allowed to feed on ICR mice anesthetized with 2% Avertin (2,2,2-Tribromoethanol, Sigma, T48402).

### Virus propagation and titration

The DAK AR41524 strain was used as a representative of the African ZIKV lineage while the SV0010/15 strain was used as a representative of the Asian ZIKV lineage. The *Aedes albopictus* cell line C6/36 (ATCC CRL-1660) was used for amplification of all virus stocks. C6/36 cells were maintained at 28°C in Leibovitz’s L-15 (L-15) medium (Sigma, L4386) with 10% fetal bovine serum (FBS, PAN BIOTECH, P30-3031), 2% tryptose phosphate broth (Sigma, T9157), 1× non-essential amino acids (Gibco, 11140-050), and 1× Pen/Strep (100 U/mL of penicillin and 100 µg/mL of streptomycin, Cytiva, SV30010).

Virus stocks were prepared in C6/36 cells according to a previously published protocol (3). After the cells were cultured to 80% confluency in 75 cm^2^ flasks, all supernatants were removed and replaced with the virus stocks at the multiplicity of infection (MOI) of 0.1 in 5 mL incomplete L-15 medium for 2 hours. After virus incubation, the supernatant was removed, replaced with 2% FBS L-15 medium, and then further incubated at 28°C. The supernatant was collected at 6–7 days post-inoculation, then supplemented with FBS to a final concentration of 20%, and stored at 80°C until further use.

The original stock of SV0010/15 was obtained from a human blood sample. The virus was initially isolated by intrathoracic inoculation of *Toxorhynchites splendens* mosquitoes (one passage) followed by propagation in the *Aedes albopictus* C6/36 cell line (three passages). The DAK AR41524 strain was initially isolated from a mosquito (*Aedes africanus*) in Kédougou, Senegal, on 17 November 1984. The original stock of DAK AR41524 obtained from BEI Resources was propagated in AP61 cells (one passage), C6/36 cells (one passage), Vero cells (two passages), and C6/36 cells (three passages). After obtaining the virus stocks from repositories, both ZIKV strains were cultured in C6/36 cells for an additional two passages in our laboratory.

Plaque assay was used to determine virus titers following a previously published protocol (7). Briefly, 100 µL of virus suspension was added to BHK-21 cells and seeded in a 24-well plate at 80% confluency. Inoculated plates were then gently rocked at room temperature for 15 minutes before incubation at 37°C, 5% CO_2_ for 45 minutes. After incubation, 1 mL of overlay medium [1% methylcellulose (Sigma, M0512) in minimal essential medium (MEM) supplemented with 2% FBS and 1× Pen/Strep] was added to each well and then further incubated at 37°C, 5% CO_2_ for 5 days. The plates were then fixed and stained with 0.5% crystal violet (Sigma, C6158) in 1:1 methanol/acetone fixative for 1 hour at room temperature. Stained plates were then washed under running tap water and air-dried before plaque counting.

### Mosquito infection by artificial membrane feeding

Mosquitoes were orally challenged with either the African or the Asian ZIKV strain using the Hemotek artificial membrane feeding system according to a previously published protocol (3). Briefly, 7-day-old female *Ae. aegypti* mosquitoes were deprived of sucrose solution overnight before being offered an artificial infectious blood meal containing 40% washed human erythrocytes and virus stock diluted to desired feeding titers for 30 minutes. After feeding, mosquitoes were anesthetized at 4°C in a refrigerator for 15 minutes, and fully engorged females were sorted on ice. Blood-fed mosquitoes were maintained in waxed paper cups with 10% sucrose solution in a climate-controlled chamber under controlled insectary conditions as mentioned above.

### Mosquito dissection for virus titration and salivation assay

Mosquitoes were cold anesthetized at 4°C in a refrigerator or on ice for 15 minutes before surface sterilization in 70% ethanol for 1 minute followed by two phosphate-buffered saline (PBS) washes. Mosquitoes were then individually dissected in drops of 1× PBS. Midgut, carcasses, and salivary glands were collected in 150 µL of MEM supplemented with 10% FBS and 1× Pen/Strep and stored at −80°C for further analysis. Tissues were homogenized using 0.5 mm glass beads with a Bullet Blender Tissue Homogenizer (NextAdvance). The 10-fold serially diluted samples were titrated by plaque assay as described above.

Mosquito saliva was collected according to a previously published protocol (3). Briefly, mosquitoes were paralyzed with triethylamine before inserting the proboscis into a pipette tip containing 20 µL of MEM supplemented with 10% FBS and 1× Pen/Strep. After 45 minutes of salivation, the media in the tips were mixed with 180 µL of MEM supplemented with 2% FBS and 1× Pen/Strep and immediately titrated by plaque assay. Mosquito salivary glands, midgut, and carcasses were dissected from anesthetized mosquitoes and stored at −80°C for virus titration by plaque assay.

### Immunofluorescent staining of ZIKV-infected midguts

Dissected midguts were cut in half in 1× PBS and washed at least twice in 1× PBS or until the blood bolus was completely removed. Blood-removed midguts were then fixed with 4% paraformaldehyde in PBS for 2 hours at room temperature and permeabilized using 2% Triton X-100 in 1× PBS for 15 minutes. After permeabilization, midguts were incubated in 2% bovine serum albumin in 1× PBS for 1 hour. Midguts were incubated with primary antibody anti-flavivirus envelope 4G2 antibody produced in-house at 4°C overnight followed by 1:500 secondary antibody anti-mouse IgG Alexa 488 (Invitrogen, A28175) in 1× PBS, and Hoechst33342 (Invitrogen, 62249) for nuclei staining for 3 hours at room temperature. Midguts were mounted onto glass slides in Vectashield Plus (Vector Laboratories, H1900). Images were taken at the same fluorescent settings for both ZIKV strains under an inverted fluorescence microscope (Olympus IX81) and laser scanning confocal microscopes (Nikon AXR and Carl Zeiss LSM900) using 20× objective lens (CFI Apochromat LWD Lambda S 20XC WI, LD Plan-Neofluar 20X/0.4 Corr M27). The z-section distance was 2.5 µm and the total z-section thickness was set between 30 and 60 µm. Z-section projection with maximum intensity was done using Fiji version 1.53t (8). The whole midgut tissues were used for cell-to-cell infection kinetics analyses.

### Infected cell count and fluorescent intensity analysis

Fiji and CellProfiler 4.2.1 (http://www.cellprofiler.org; Broad Institute, Cambridge, MA, USA) (9) were used for image analysis. In Fiji, grayscale images of infected cells and nuclei proceeded to background subtraction and object segmentation to generate binary images using the following plugins: Trainable Weka Segmentation (TWS) (10) for infected cells segmentation and StarDist 2D (11) for nuclei segmentation. Watershed and segmented line tools in Fiji were applied to binary images of infected cells generated from TWS to separate individual cells.

Workflows in CellProfiler were created to count the number of infected foci, identify the number of cells in infected foci, and measure the fluorescent intensity of infected cells and their nuclei. To count the number of infected cells in foci, binary images of infected foci and nuclei were used as input images for identifying primary objects. To measure fluorescent intensity, binary images of individual infected cells and nuclei were used as input images. A mask of infected foci/cells was used for extracting the nuclei of infected cells. Additional details on the image analysis can be found in Fig. S1. Fluorescent intensity of 4G2 staining was measured from maximum Z-projected images of infected midguts taken with confocal microscopes with the 20× objective lens. Only infected cells from the midgut monolayer and non-overlapping infected cells from the midgut multilayer were measured.

### Statistical analyses

Statistical analyses in this study were conducted using the rstatix package (version 0.7.1) (12) in R (version 4.3.0). Each data set was tested for normality using the Shapiro-Wilk test for Normality. The statistical significance of differences between the two groups was addressed using a *t*-test for normally distributed data or the Wilcoxon rank-sum test for non-normally distributed data. Multiple comparison of non-normally distributed data was conducted using Kruskal-Wallis followed by Dunn’s post hoc test. Graphs were generated using the ggpubr package (version 0.6.0) (13) in R.

## RESULTS

### The African ZIKV strain displays faster intra-host infection kinetics than the Asian ZIKV strain

To identify intra-host infection barriers that lead to different transmissibility between African and Asian ZIKV lineages, we compared infection kinetics of two representative strains during midgut infection, dissemination (virus escape from the midgut and systemic infection), salivary gland infection, and release in saliva over time. A laboratory strain of Thai *Ae. aegypti* was fed with approximately 7 log_10_ PFU/mL of African (DAK AR41524) or Asian ZIKV (SV0010/15). Uncontrolled variation in infectious dose was less than 0.2 log_10_ PFU/mL across experiments and ZIKV strains.

**Fig 1:**
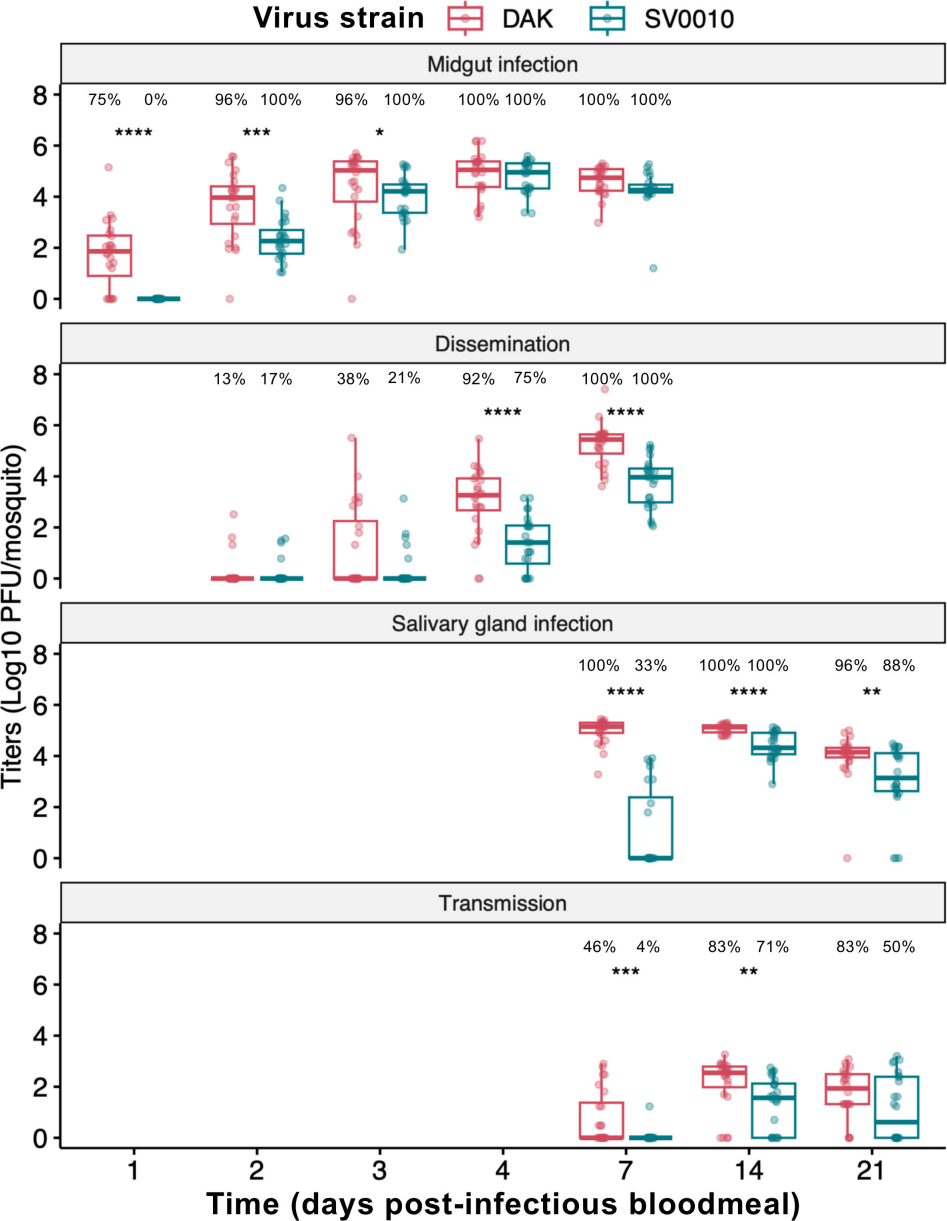
African ZIKV has more rapid intra-host infection kinetics than Asian ZIKV. Intra-host infection kinetics of African (DAK) and Asian (SV0010) ZIKV strains in *Ae. aegypti*. The midgut infection was investigated at 1, 2, 3, 4, and 7 dpibm. Dissemination (escape of virus from the midgut and replication in the body) was measured in the carcasses at 2, 3, 4, and 7 dpibm. Salivary gland infection and transmissibility (virus titers in saliva) were measured at 7, 14, and 21 dpibm. Data were summarized from two blood-feeding experiments with at least 10 mosquitoes from each. The feeding titers for the midgut infection and dissemination samples were 7.2–7.38 log_10_ PFU/mL for DAK and 7.34–7.38 log_10_ PFU/mL for SV0010. Box and scatter plots represent the distribution of viral titers in log_10_-transformed PFU/mosquito. Statistical analysis comparing infection levels was conducted using Kruskal-Wallis followed by Dunn’s post hoc test in R. *P < 0.05, **P < 0.01, ***P < 0.001, ****P < 0.0001. The percentage on top of each graph indicates infection prevalence (number of samples with positive virus x 100/number of total sample).

The infection kinetics revealed that African ZIKV was more efficient at establishing midgut infection than Asian ZIKV (Fig. 1). Over 50% of mosquitoes fed with African ZIKV had a detectable infectious virus in their midguts at 1 day post-infectious blood meal (dpibm), while no detectable infection was observed for Asian ZIKV. The observed variations in African and Asian ZIKV titers at 1 dpibm cannot be attributed to differences in the residual infectious virus within the blood bolus, as both strains exhibited similar rates of decline in infectious virus levels during the early timepoints (Fig. S2). Although midgut viral titers of the African strain remained higher than those of the Asian strain at 2–3 dpibm, midgut infection level eventually plateaued, and the Asian strain reached comparable titers to the African strain from 4 dpibm onward.

During the subsequent phase of intra-host infection kinetics, similar dissemination prevalence (% of mosquitoes with detectable virus outside the midgut) at early timepoints was observed for both strains, suggesting that midgut escape may not be a bottleneck in the Asian ZIKV transmission cycle (Fig. 1). However, African ZIKV displayed significantly higher dissemination titers at 4 dpibm, and this trend persisted through 7 dpibm. The higher virus titers of African ZIKV during later timepoints of dissemination might be due to a higher number of viruses available to escape the midgut to initiate a systemic infection, or a higher replication rate of the African ZIKV strain in secondary organs compared to the Asian ZIKV strain.

Next, we investigated salivary gland infection and transmissibility of the viruses at 7, 14, and 21 dpibm. We found that the African ZIKV strain reached the salivary glands earlier and replicated more efficiently than the Asian ZIKV strain (Fig. 1). African ZIKV reached the salivary glands of all mosquitoes by 7 dpibm, while Asian ZIKV was only detected in the salivary glands of 33% of blood-fed mosquitoes. Although salivary glands of all blood-fed mosquitoes with Asian ZIKV became infected by 14 dpibm, average virus titers of African ZIKV were higher than those of Asian ZIKV across all time points measured suggesting a more robust virus propagation in salivary gland tissue.

African ZIKV could be detected in saliva from 46% of mosquitoes at 7 dpibm while only one out of 24 of the Asian ZIKV blood-fed mosquitoes had a detectable infectious virus in saliva (Fig. 1). The prevalence and titers in the saliva of both viruses increased from 7 to 14 dpibm. Interestingly, the saliva virus titers at 21 dpibm were lower than 14 dpi for both viruses suggesting lower transmissibility at the very late timepoint. These results confirm the higher transmissibility of the African ZIKV strain than the Asian ZIKV strain in *Ae. aegypti* in our study system. Taken together, our results suggested that the higher transmissibility of the African ZIKV strain was a result of a more rapid infection starting from the midgut infection, dissemination, and salivary gland infection. The lower transmissibility of the Asian ZIKV strain may therefore be caused by the lower amount of virus that is available to escape salivary glands given the lower prevalence and titers of salivary gland infection.

Infection kinetics in all mosquito tissues revealed that the key feature of the African ZIKV strain was a higher replication rate in infected cells in various tissue types prior to salivary gland escape. However, it remains unclear whether the increased replication is due to higher virus production in individual cells, faster viral spread within tissues, or both.

The results shown in Fig. 1 suggest that the replication kinetics of two ZIKV strains can be readily differentiated during the establishment of midgut infection. Our experimental approach allows the initial viral inoculum to be controlled (using the same titer in the blood meal) so that differential cell-to-cell spread can easily be observed in the intact tissue. We next investigated the cell-to-cell level infection kinetics in the midgut by focusing on the three critical steps for successful midgut infection: (i) the establishment of virus infection in midgut epithelial cells (primary infection), (ii) the replication of virus in the primary infected cells, and (iii) the spread of virus from the primary infected cells to neighboring cells (secondary infection).

### African ZIKV exhibits superior primary midgut infection success compared to Asian ZIKV

**Fig 2:**
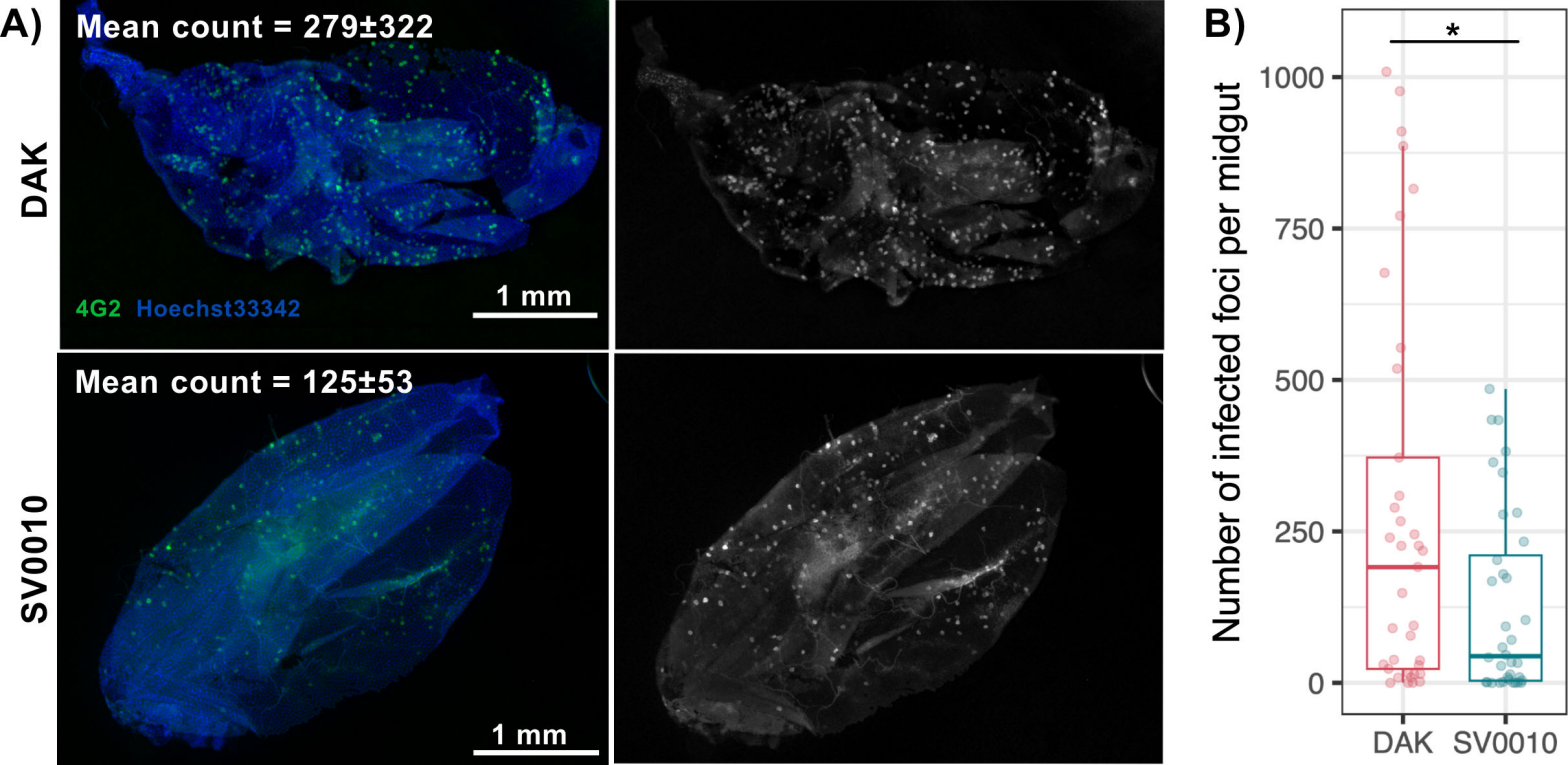
African ZIKV establishes midgut infection better than Asian ZIKV. (Left) Representative image of *Ae. aegypti* midguts infected by the Asian ZIKV strain (SV0010) or the African ZIKV strain (DAK) at 1 dpibm. ZIKV was stained using the 4G2 primary antibody followed by a secondary goat anti-mouse antibody conjugated with Alexa Fluor 488. Nuclei were stained with Hoechst 33342. (Right) Box and scatter plot comparing number of infected cell foci in each midgut. Each dot represents data from an individual midgut. Data were summarized from two blood-feeding experiments with at least 10 midguts from each. The feeding titers for the midgut infection and dissemination samples were 7.2–7.38 log_10_ PFU/mL for DAK and 7.34–7.38 log_10_ PFU/mL for SV0010. Statistical analysis was conducted using the Wilcoxon rank-sum test, *P < 0.05.

To compare the infectivity of African and Asian ZIKV during primary infection of the mosquito midgut, we compared the number of midgut cells that became infected after ingestion of an infectious blood meal containing approximately 7 log_10_ PFU/mL of each ZIKV strain. Infected cells in the midgut at 1 dpibm were visualized using immunofluorescent staining. The number of infected cell foci for the African ZIKV strain was more than twice that of the Asian ZIKV strain, with a mean number of 279 ± 322 and 125 ± 53 infection foci per midgut, respectively (Fig. 2). These results indicate that, given similar blood meal titers, African ZIKV establishes midgut infection more effectively than Asian ZIKV.

### Midgut cells infected with African ZIKV display stronger immunofluorescence staining than those with Asian ZIKV

**Fig 3:**
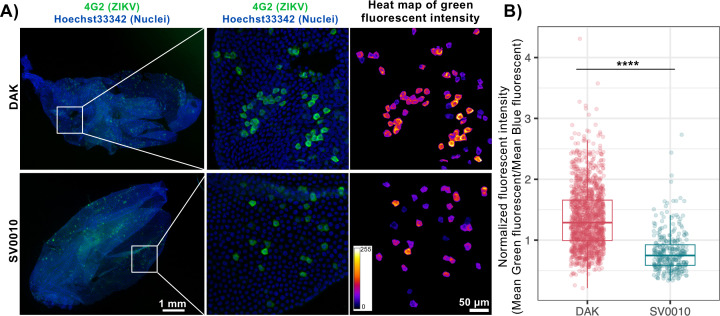
Midgut cells infected with African ZIKV display stronger immunofluorescence signals than those with Asian ZIKV. (**A**) Representative images of *Ae. aegypti* midguts infected by the Asian ZIKV strain (SV0010) or the African ZIKV strain (DAK) ZIKV at 1 dpibm. The overall midgut images were epifluorescence images under a 4× objective lens the same images as those in Fig. 2A. The zoomed images were maximum projected images of confocal z-stacks under a 20× objective lens. ZIKV was stained using the 4G2 primary antibody followed by a secondary goat anti-mouse antibody conjugated with Alexa Fluor 488. Nuclei were stained with Hoechst 33342. The heatmaps demonstrate the green channel fluorescence intensity on a scale ranging from 1 to 255 arbitrary units. (**B**) Box and scatter plots comparing the immunofluorescence intensity of each infected cell between the two ZIKV strains. Whole mosquito midguts, the same samples as in Fig. 2, were used for immunofluorescence intensity analyses. The normalized fluorescence intensity was calculated by dividing the mean Alexa 488 fluorescence intensity of each infected cell with the mean Hoechst 33342 fluorescence intensity of its nucleus. Because the tissue can be folded or have multiple layers in the *in situ* analysis, infected cells in the areas having overlapped infected regions were excluded to avoid inaccurate evaluation of the fluorescence intensity of each cell. Each dot represents data from an individual infected cell. Data were summarized from two blood-feeding experiments with at least 5 midguts from each. The feeding titers for the midgut infection and dissemination samples were 7.2–7.38 log_10_ PFU/mL for DAK and 7.34–7.38 log_10_ PFU/mL for SV0010. Statistical analysis was conducted using the Wilcoxon rank-sum test, ****P < 0.0001.

To further investigate ZIKV infection dynamics, we assessed the intensity of immunofluorescence staining in each infected cell (Fig. 3). In total, we examined 1,417 infected cells for the African ZIKV strain and 318 infected cells for the Asian ZIKV strain. Our results showed that the normalized immunofluorescence intensity in midgut cells infected by the African ZIKV strain was significantly higher than that in cells infected by the Asian ZIKV strain (mean normalized signals of 1.36 ± 0.50 and 0.80 ± 0.33, respectively, *P* < 0.0001, Fig. 3B). The stronger immunofluorescence staining in infected cells suggests that at 1 dpibm, a larger amount of ZIKV E proteins were produced in cells infected with the African strain compared to those infected with the Asian strain. This could be attributed to either a higher level of virus production per cell or a more rapid cellular replication cycle.

### African ZIKV spreads to neighboring midgut cells faster than Asian ZIKV

**Fig 4:**
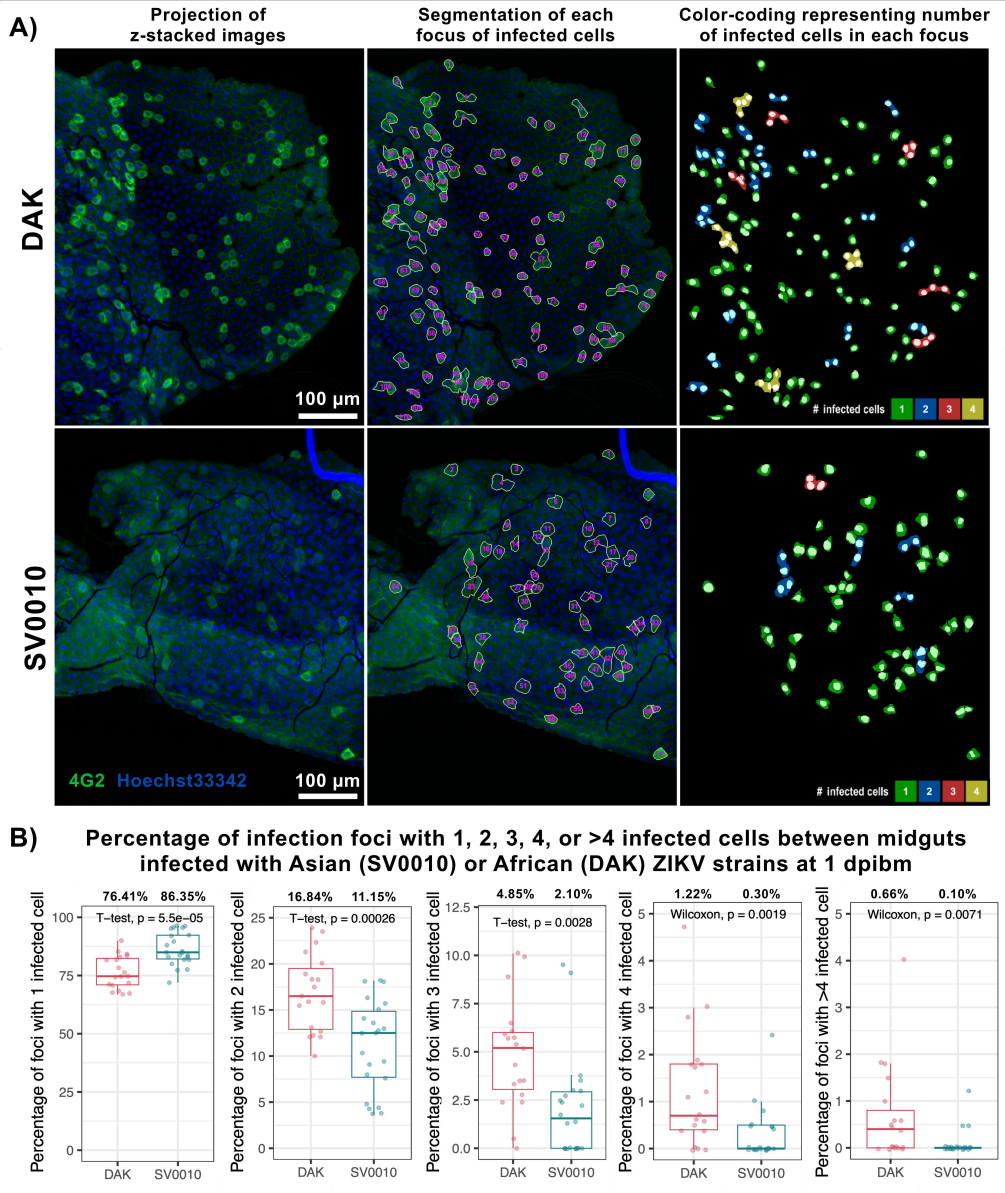
African ZIKV has more foci with secondary infection than Asian ZIKV. (**A**) Representative images demonstrating the infected cell counting. ZIKV was stained using the 4G2 primary antibody followed by a secondary goat anti-mouse antibody conjugated with Alexa Fluor 488. Nuclei were stained with Hoechst 33342. The midguts used in the analyses were the same samples as in Fig. 2. (Left) Projection of representative z-stacked images. (Middle) Segmentation of each focus of infected cells. Some areas of the midgut were folded on top of each other thus having more than one layer of epithelial cells. (Right) Color-coding representing a number of infected cells in each focus. (**B**) Box and scatter plots comparing the percentage of infection foci with 1, 2, 3, 4, or >4 infected cells between midguts infected with Asian (SV0010) or African (DAK) ZIKV strains at 1 dpibm. Data were summarized from two blood-feeding experiments. The feeding titers for the midgut infection and dissemination samples were 7.2–7.38 log_10_ PFU/mL for DAK and 7.34–7.38 log_10_ PFU/mL for SV0010. Each dot represents data from an individual midgut. Statistical analysis was conducted using a Student *t*-test or Wilcoxon rank-sum test.

The later stage of tissue-level infection kinetics during midgut infection involves the spread of infection from primary infected cells to neighboring cells (secondary infection). In this experiment, we compared the rate of virus spread in the midgut tissue by measuring the size of infection foci (number of infected cells in each infection focus) at 1 dpibm. With blood meal titers of 7 log_10_ PFU/mL, we observed that at 1 dpibm, mosquito midguts infected with African ZIKV displayed a lower percentage of infection foci containing only a single infected cell (primary infection) compared to Asian ZIKV (76.41% and 86.35%, respectively; Fig. 4). Notably, the number of foci with more than one infected cell (2, 3, 4, and >4 infected cells per focus) was significantly higher for the African strain than for the Asian strain, indicating faster secondary infection and a greater spread to neighboring cells of the African strain (Fig. 4).

**Fig 5:**
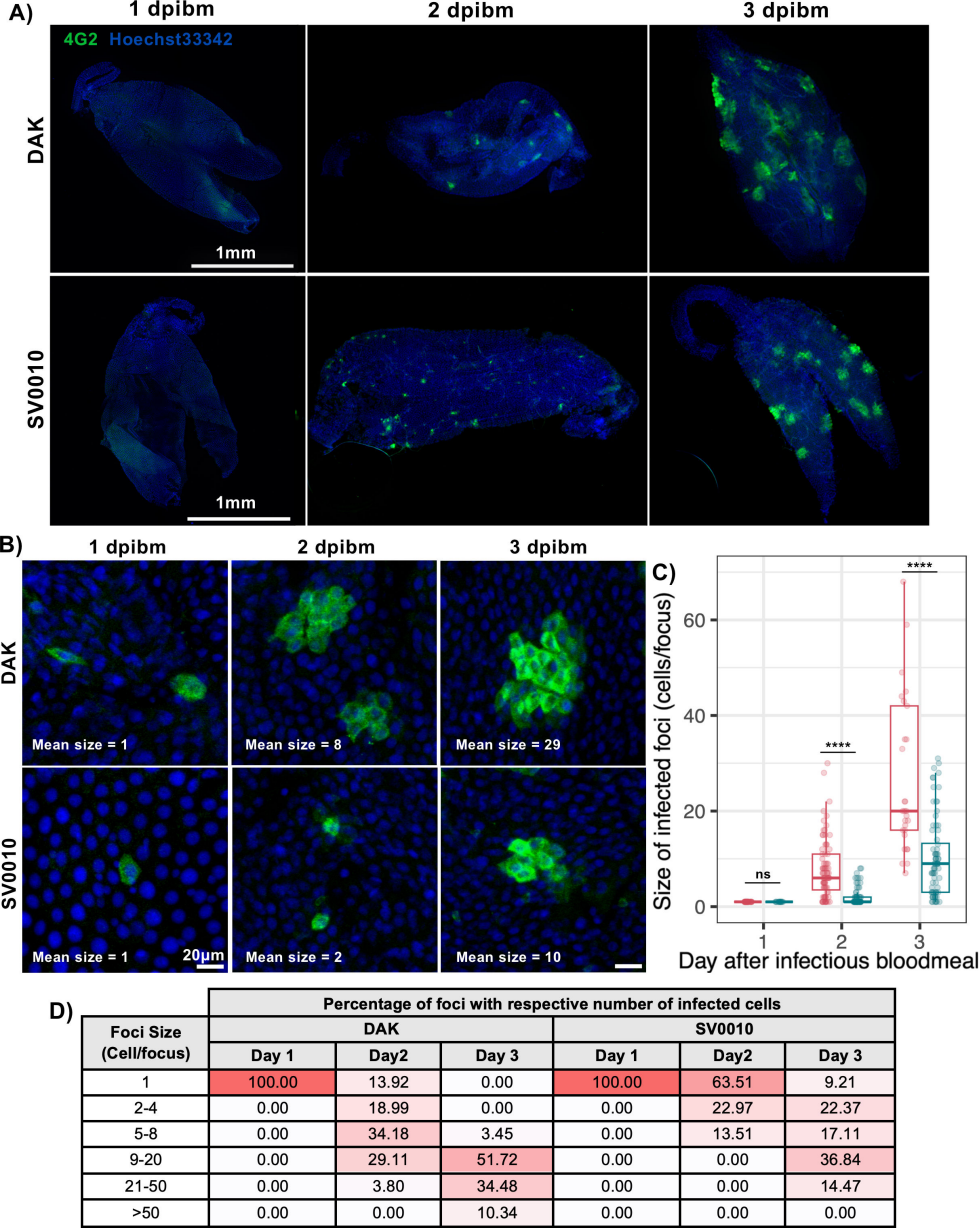
African ZIKV spreads faster to neighboring cells than Asian ZIKV. (**A**) Overall images of *Ae. aegypti* midguts infected with 5 log_10_ PFU/mL of the African ZIKV strain (DAK) or the Asian ZIKV strain (SV0010) at 1, 2, and 3 dpibm. ZIKV was stained using the 4G2 primary antibody followed by a secondary goat anti-mouse antibody conjugated with Alexa Fluor 488. Nuclei were stained with Hoechst 33342. (**B**) Higher magnification representative images (200×) demonstrating the size of infection foci. (**C**) Box and scatter plots demonstrating the number of cells in each infection focus of African and Asian ZIKV. The feeding titers for the midgut infection and dissemination samples were 4.79–4.84 log_10_ PFU/mL for DAK and 5.14–5.25 log_10_ PFU/mL for SV0010. Statistical analysis was conducted using the Kruskal-Wallis test followed by Dunn’s post hoc test in R, ****P < 0.0001. (**D**) Table comparing the distribution of infection focus sizes over time during 1, 2, and 3 dpibm.

In addition to comparing the size of infection foci at 1 dpibm, we conducted an experiment to examine the expansion of infection foci over time (Fig. 5). Given that blood meal titers of 7 log_10_ PFU/mL resulted in hundreds of primary infected cells, measuring the expansion of infected cell foci during subsequent timepoints with such a high number of primary infections was infeasible (Fig. S3). Therefore, blood meal titers of approximately 5 log_10_ PFU/mL were used to infect *Ae. aegypti* in this experiment. To measure the expansion of infection foci, mosquito midguts were collected at 1, 2, and 3 dpibm for immunofluorescence staining. We quantified the number of infected cells within infection foci and calculated the percentage of foci containing 1, 2–4, 5–8, 9–20, 21–50, or ≥50 infected cells per focus. Interestingly, with the blood meal titers of 5 log_10_ PFU/mL, none of the infected foci had any secondary infection for either African or Asian ZIKV on the first day (Fig. 5). This is in contrast with the results obtained using higher blood meal titers of 7 log_10_ PFU/mL, which resulted in secondary infection observed at 1 dpibm (Fig. 4). Nonetheless, we found that African ZIKV spread to neighboring cells more rapidly than Asian ZIKV. At 2 dpibm, only 13.92% of midgut foci had a primary infection (one infected cell in each focus) for the African ZIKV strain, while midguts still had a primary infection in 63.51% of foci for the Asian ZIKV strain. By 3 dpibm, all African ZIKV-infected foci exhibited secondary infection with at least 5 cells per foci, while Asian ZIKV-infected foci still had primary infection in 9.21% of cases and secondary infection in 22.37% of foci with fewer than 4 cells. The faster spread of African ZIKV resulted in larger average infected focus sizes during 2–3 dpibm (Fig. 5D). The African ZIKV and Asian ZIKV had average focus sizes of 8 ± 6 and 2 ± 2 infected cells per focus at 2 dpibm, and 10 ± 8 and 29 ± 22 infected cells per focus at 3 dpibm, respectively.

### Progression of ZIKV infection in the midgut tissue varies between different midgut cell populations

**Fig 6:**
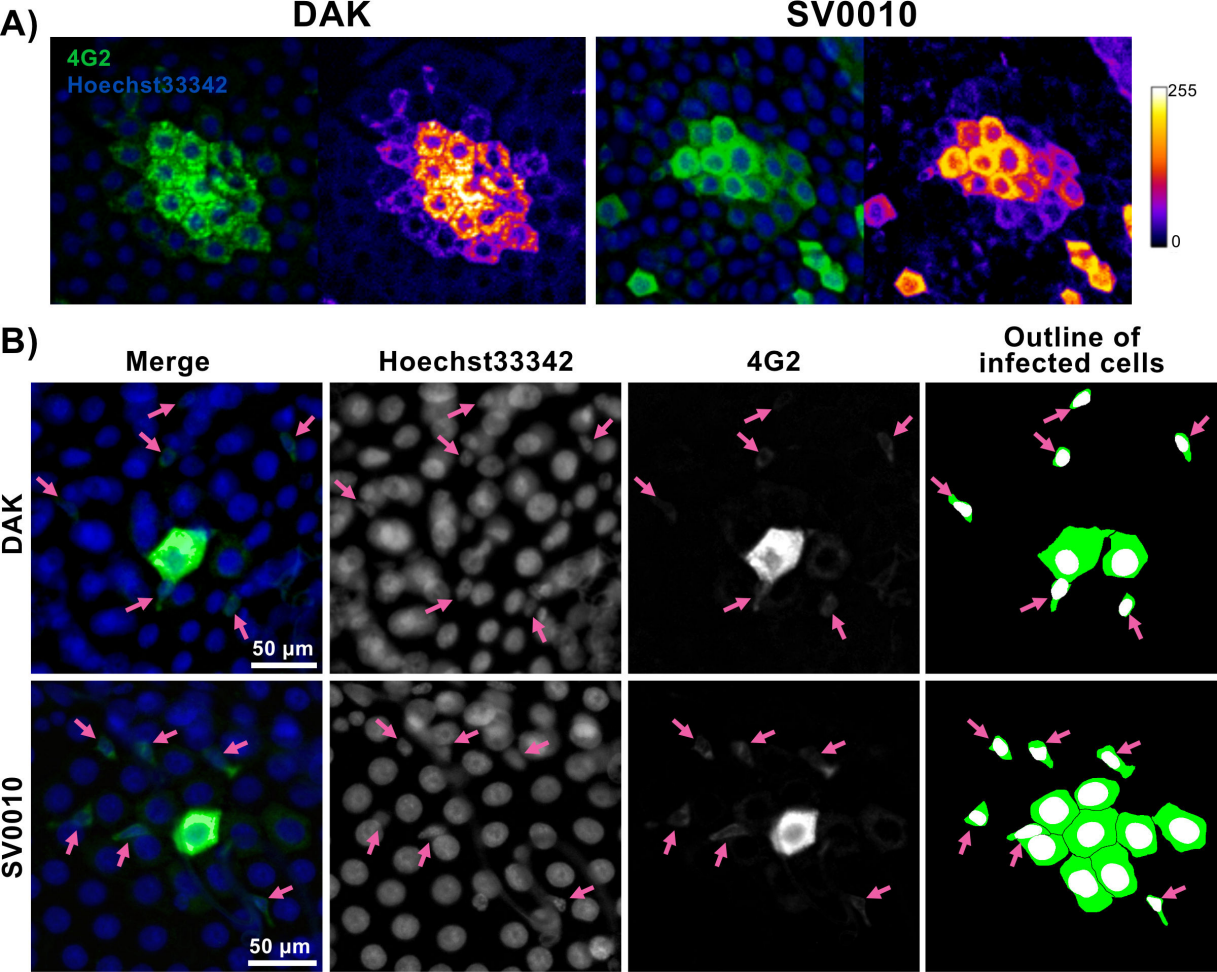
Progression of ZIKV infection in midgut cells. (**A**) Immunofluorescence staining of infected cell clusters with secondary infections. Immunofluorescence images of the African ZIKV strain (DAK) and the Asian ZIKV strain (SV0010). ZIKV was stained using the 4G2 primary antibody followed by a secondary goat anti-mouse antibody conjugated with Alexa Fluor 488. Nuclei were stained with Hoechst 33342. The heatmap demonstrates the green channel fluorescence intensity on a scale ranging from 1 to 255 arbitrary units. (**B**) Infection foci of both ZIKV strains at 2 dpibm. Pink arrowheads indicate infection in small cells of the focus periphery.

The midgut tissue consists of distinct cell populations with varying morphologies and functions. Based on previous research, cells with large cytoplasm and nuclei are likely enterocytes (ECs) while cells with smaller nuclei are likely enteroendocrine cells (EEs), and undifferentiated progenitors including intestinal stem cells (ISCs) and enteroblasts (EBs) (14, 15). We observed that all the primary infected cells are likely ECs as they have large nuclei and cytoplasm. As the infection progressed, we observed cells with intense immunofluorescence staining at the center of each infection focus. These cells were surrounded by cells of similar size but with a subtle decrease in immunofluorescence intensity along the radial distance from the center cells (Fig. 6A). This indicates that the infection gradually spreads from the primary infected cells to neighboring ECs. Interestingly, in some infected foci, we observed the infection in small satellite cells with small nucleus (Fig. 6B). The small infected cells are on the periphery of the foci but usually not immediately adjacent to the infected ECs.

## DISCUSSION

Although it is well established that the African ZIKV lineage exhibits higher transmissibility in *Aedes* mosquitoes compared to the Asian ZIKV lineage (3, 16–18), limited knowledge exists regarding ZIKV infection kinetics during the early steps of mosquito infection and the virus’s interaction with various mosquito tissues. To enhance our understanding of the differential transmissibility of the two ZIKV lineages, our study compared the intra-host tissue-level infection kinetics of African and Asian ZIKV strains in *Ae. aegypti* mosquitoes by examining both the infection intensity (amount of infectious virus as measured by plaque assay) and prevalence during midgut infection, dissemination, salivary gland infection, and virus release in saliva over time. We observed that the African ZIKV strain established a midgut infection, disseminated systemically, and was released in saliva more rapidly than the Asian ZIKV strain.

We then utilized *in situ* immunofluorescence staining to investigate cell-to-cell infection kinetics and pinpoint differential infection barriers between the two ZIKV strains. Although previous studies have employed similar immunofluorescence staining techniques to visualize the progression of arbovirus infection in mosquito tissues (19, 20), these studies lacked a quantitative assessment of infection, hindering a comprehensive comparison of infection kinetics. By quantitatively analyzing cell-to-cell infection kinetics, we were able to provide insights into host-virus interactions at a higher resolution, contributing valuable information on the differential infection kinetics between the two ZIKV lineages. The key feature of African ZIKV is a more robust virus propagation which may be attributed to increased virus production in individual cells, faster viral spread within tissues, or both. In the midgut tissue, the African ZIKV strain established primary infections in the midgut epithelial cells, replicated in the primary infected cells and spread throughout the mosquito tissue more efficiently than the Asian ZIKV strain.

Combining infectious virus detection by plaque assay and *in situ* immunofluorescence staining of the midgut, our data suggest that African ZIKV replicates at a faster rate than Asian ZIKV. At 1 dpibm, infectious Asian ZIKV could not be detected in any mosquito by plaque assay despite positive immunofluorescence signals in the midgut, suggesting either incomplete viral assembly within the first day or infectious titers below the limit of detection by plaque assay. Conversely, at the same time point, half of the African ZIKV-fed mosquitoes exhibited detectable infectious viruses, indicating a more robust propagation. The eclipse phase of Asian ZIKV replication in the midgut aligns with previous findings with the I-44 Mexican ZIKV strain (19). The faster replication of African ZIKV was also observed in mammalian cells such as human neural stem cells, primary human astrocytes, Vero (African green monkey kidney), HEK-293 (human embryonic kidney), and RK-13 (Rabbit kidney) cell lines (21–23) as well as several insect cell lines (23) suggesting that the phenotype is conserved across mammalian and insect hosts (reviewed in reference 24).

Using immunofluorescence staining of midguts at an early timepoint, we could estimate the number of primary infection foci. This analysis demonstrated a major bottleneck during the establishment of midgut infection shared by African and Asian ZIKV. With blood meal titers of 7 log_10_ PFU/mL, each mosquito is expected to ingest 10,000–20,000 PFU (1–2 µL of blood meal), yet the majority of midguts had a number of primary infected cells in the range of hundreds, meaning that only a few percent of infectious virions successfully establish a midgut infection. The scale of the bottleneck and the size of the founding population are similar to previous estimates based on sequencing approaches for the dengue virus (25, 26), and immunofluorescence staining-based techniques for dengue virus (20, 27) and Venezuelan equine encephalitis infectious clones (28).

Because *in situ* immunofluorescence staining preserves spatial information of virus spread in mosquito tissues, the technique can be used to investigate characteristics of virus infection in different cell types, in addition to the rate of cell-to-cell spread. We observed that midgut cells with small nuclei support faster ZIKV replication than midgut cells with large nuclei. These observations provide several insights into the cell-to-cell virus progression. Because the immunofluorescence staining requires a certain level of viral protein accumulation in the cells, the technique is expected to preferentially detect late stages of infection. Therefore, by the time the infection is detectable in the primary infected cells, the secondary infections already occur in the neighboring cells. We speculate that the small satellite-infected cells might have a faster replication cycle than the cells with large nuclei; thus, the immunofluorescence signal was detected earlier. Alternatively, the brighter immunofluorescence signals in the small cells might result from the more concentrated viral protein due to the smaller size of their cytoplasm. This observation suggests that different cell populations have a different ability to support virus replication. The identity of these small midgut cells has yet to be determined due to a lack of tools for identifying cell types, such as antibodies or transgenic reporter lines. A recent single-cell transcriptomics study that identified markers for each midgut cell population will make these molecular tools feasible in the future (29).

Another intriguing finding from our time-course analysis is that ZIKV infection in midguts exposed to a higher blood meal titer (7 log_10_ PFU/mL) progressed more quickly than in midguts exposed to a lower blood meal titer (5 log_10_ PFU/mL). Although it may seem logical that a larger virus inoculum would result in a more productive infection, our results demonstrated that a higher blood meal titer did not only result in a greater number of virus particles initiating the infection but also a faster replication and spread in the mosquito tissues. This implies that the midgut responses differ according to the inoculum size. This is consistent with a previous study on dengue virus (30), which observed that a lower blood meal titer resulted in lower viral loads in mosquito tissues even during the crossing of subsequent infection barriers at later timepoints. Several host machinery including those of glucose metabolism, lipid/phospholipid metabolisms, cell proliferation, apoptosis, and immunity have been shown to influence virus infection in mosquitoes (31–36). It is possible that a higher amount of virus initiating the infection could manipulate these host machineries at a larger scale in mosquito tissues and thereby enhance virus replication. Since most arboviruses establish persistent infections in mosquito vectors, which are characterized by steady-state dynamics between virus and host, a strong manipulation of host machinery early on might provide an edge to the virus and allow higher replication during persistent infection.

Our findings of differential intra-host infection kinetics contribute to a better understanding of the differences in transmissibility between African and Asian ZIKV strains. Further investigation into the viral and host factors driving the differences in propagation efficiency between these two ZIKV lineages is essential for unraveling the underlying mechanisms and informing the development of ZIKV transmission control strategies. Advanced techniques such as CRISPR/Cas9 genome editing, proteomics, and single-cell RNA sequencing can be employed in future studies to provide more insights into the molecular determinants of the observed strain-specific infection kinetics.

## Data Availability

The data and immunofluorescence images from this study are available upon request to the corresponding author.

## SUPPLEMENTAL MATERIAL

Figure S1 msphere.00545-23-s0001.tif Detection of infected foci/cells and their nuclei.

Figure S2 msphere.00545-23-s0002.tif Stability of infectious virus titers in *A. aegypti* midguts during early time points was similar between African and Asian ZIKV.

Figure S3 msphere.00545-23-s0003.tif Representative images of infection kinetics at 1, 2, and 3 dpibm of midguts infected by 7 log10 PFU/mL of the African ZIKV strain (DAK) and the Asian ZIKV strain (SV0010).

ASM does not own the copyrights to Supplemental Material that may be linked to, or accessed through, an article. The authors have granted ASM a non-exclusive, world-wide license to publish the Supplemental Material files. Please contact the corresponding author directly for reuse.
