# Supplementary figures and images for: Differential intra-host infection kinetics in Aedes aegypti underlie superior transmissibility of African relative to Asian Zika virus
Source: mSphere. 2023 Nov 9;8(6):e00545-23. doi: 10.1128/msphere.00545-23 (PMC10732021; doi:10.1128/msphere.00545-23)

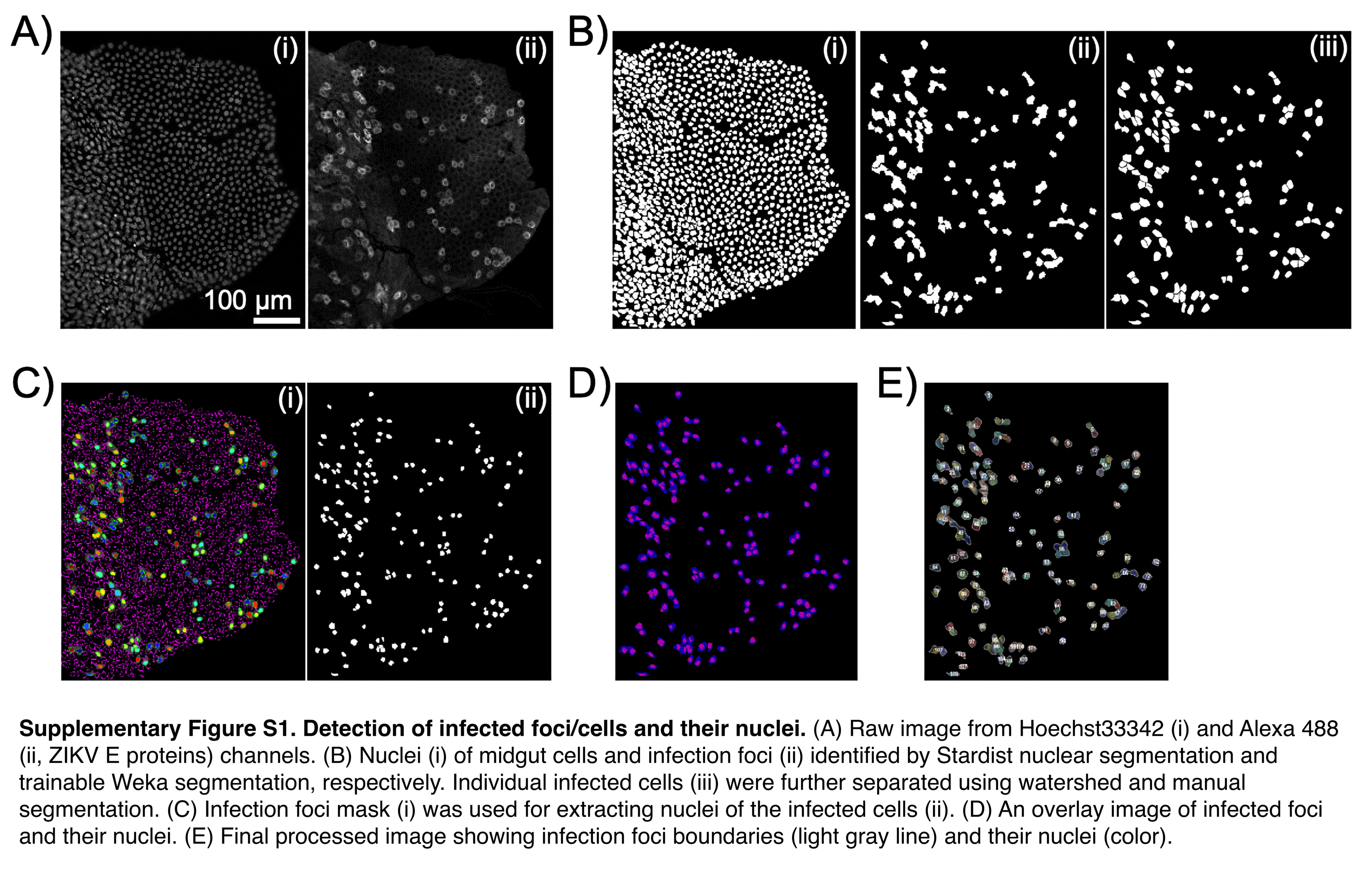

Supplement: Figure S1 — Detection of infected foci/cells and their nuclei. [file msphere.00545-23-s0001.tif]

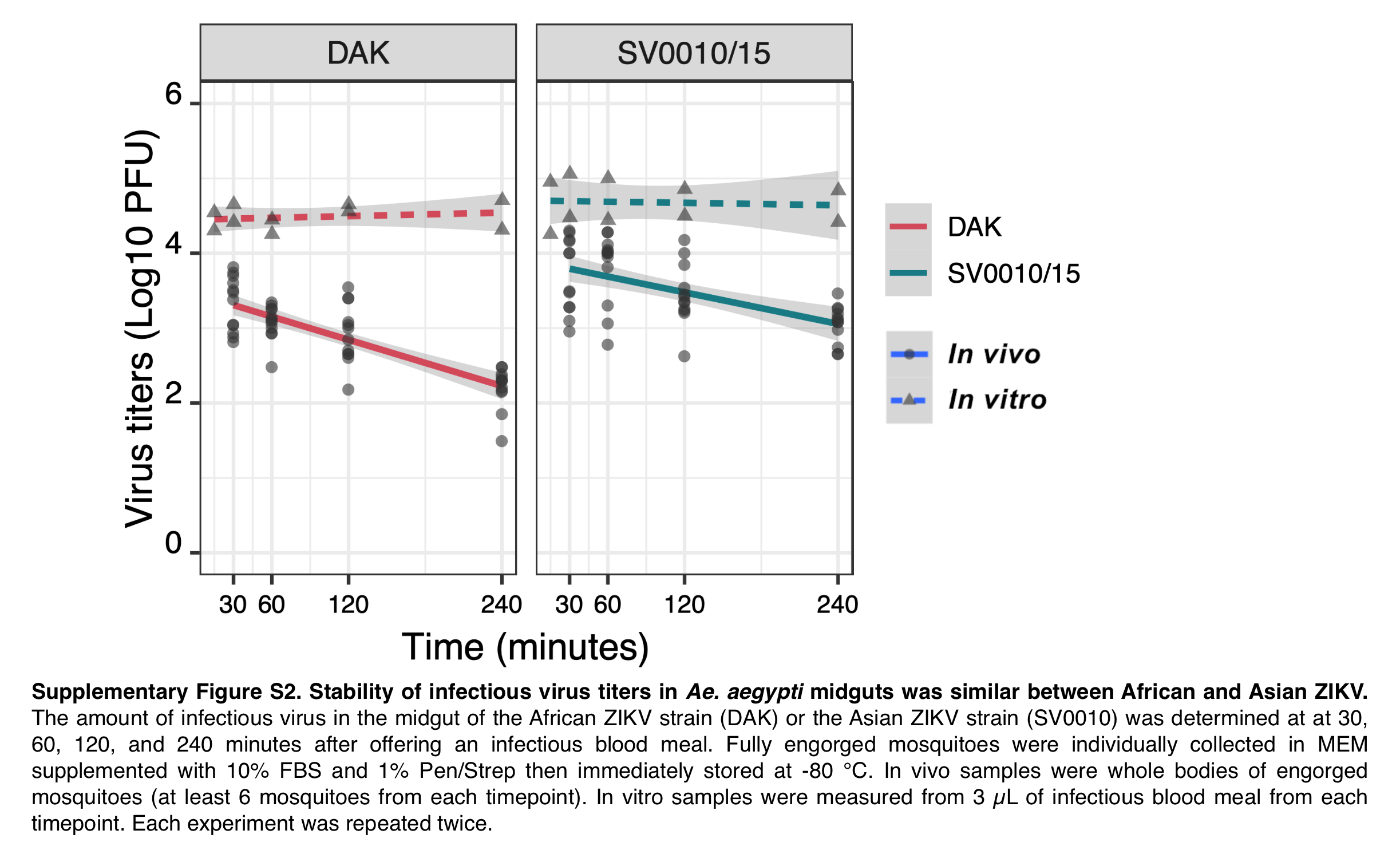

Supplement: Figure S2 — Stability of infectious virus titers in A. aegypti midguts during early time points was similar between African and Asian ZIKV. [file msphere.00545-23-s0002.tif]

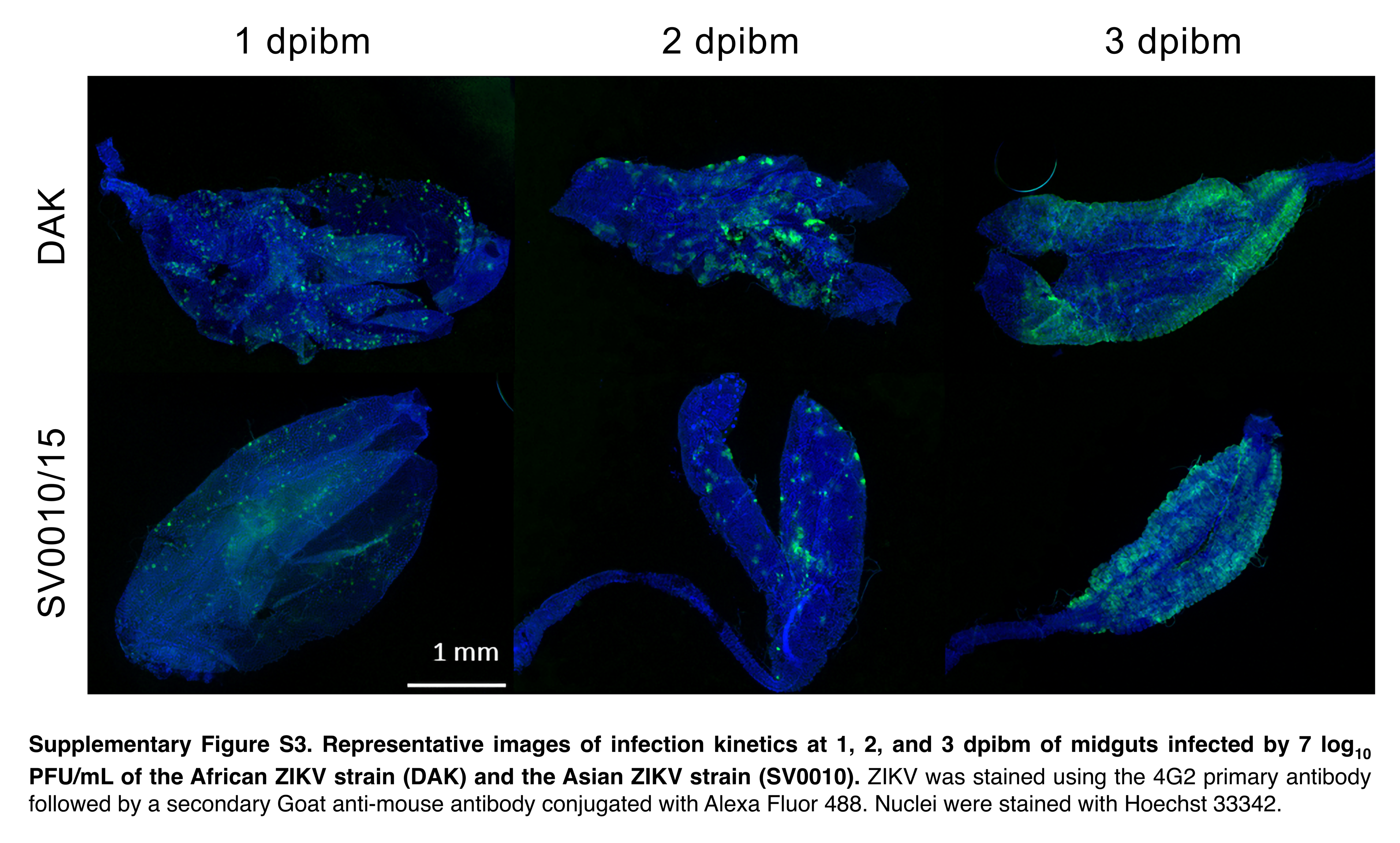

Supplement: Figure S3 — Representative images of infection kinetics at 1, 2, and 3 dpibm of midguts infected by 7 log10 PFU/mL of the African ZIKV strain (DAK) and the Asian ZIKV strain (SV0010). [file msphere.00545-23-s0003.tif]
